# Radiation Resistance and Adsorption Behavior of Aluminum Hexacyanoferrate for Pd

**DOI:** 10.3390/toxics11040321

**Published:** 2023-03-29

**Authors:** Yueying Wen, Yan Wu, Lejin Xu

**Affiliations:** 1 School of Nuclear Science and Engineering, Shanghai Jiao Tong University, Shanghai 200240, China; 2 School of Energy and Power Engineering, Huazhong University of Science and Technology, Wuhan 430074, China

**Keywords:** γ irradiation, aluminum hexacyanoferrate, palladium, adsorption site

## Abstract

Irradiation resistance is important for adsorbents used in radioactive environments such as high-level liquid waste. In this work, a silica-based composite adsorbent (KAlFe(CN)_6_/SiO_2_) was synthesized and γ-irradiated from 10 to 1000 kGy. The angles of the main X-ray diffraction peaks slightly decreased with the increase in irradiation dose, and a minor decomposition of CN^−^ occurred after irradiation to 1000 kGy, indicating that the KAlFe(CN)_6_/SiO_2_ adsorbent could preserve structural integrity with a dose below 100 kGy. In 1 to 7 M HNO_3_, the adsorption ability of the irradiated KAlFe(CN)_6_/SiO_2_ remained performant, with a higher *K*_d_ than 1625 cm^3^ g^−1^. The adsorption equilibrium of Pd(II) in 3 M HNO_3_ was attained within 45 min before and after irradiation. The maximal adsorption capacity *Q*_e_ of the irradiated KAlFe(CN)_6_/SiO_2_ on Pd(II) ranged from 45.1 to 48.1 mg g^−1^. A 1.2% relative drop in *Q*_e_ was observed after 100 kGy irradiation, showing that γ-irradiation lower than 100 kGy insignificantly affected the adsorption capacity of KAlFe(CN)_6_/SiO_2_. Calculating and comparing the structures and free energies of different adsorption products via the density functional theory (DFT) method showed that KAlFe(CN)_6_/SiO_2_ was more inclined to completely adsorb Pd(II) and spontaneously generate Pd[AlFe(CN)_6_]_2_.

## 1. Introduction

The development of nuclear energy has brought evident benefits in reducing carbon emissions and promoting human health [1,2], although the disposal of spent highly radioactive fuel discharged from nuclear power plants is complex and challenging [3,4,5] partly because of the limited miscibility of platinum group metals (PGMs) [6]. The plutonium uranium extraction (PUREX) process is commonly used to reprocess spent fuel and produces approximately 500 L of radioactive high-level liquid waste (HLLW) per ton of spent fuel. HLLW is dangerous, and has a complex chemical composition and radiation levels of up to 1000 Ci/L [7]. If HLLW is released into the biosphere, radioactive and heavy-metal elements may migrate to the soil and plants, destroying the genetic material of organisms and human organs [8,9]. Therefore, the proper disposal of HLLW, such as by separating high-heat radionuclides, is necessary to protect humans and the biosphere [10,11,12].

Glass solidification is widely used to form a solidified product for HLLW that can envelop radioactive elements and reduce waste volume. This is a type of technology that dissolves the ions in HLLW into amorphous borosilicate glass or phosphate glass to form a new, stable structure. Some studies indicated that the separation of Pd is beneficial for the safety of glass solidification [13,14,15]. Palladium has a low solubility of 0.3 mg g^−1^ in glass, facilitating precipitation during glass solidification [16]. The precipitated substance has high density, viscosity, and electric conductivity, leading to problems such as blocking the outlet and short-circuiting the electrodes in the melter, threatening production safety. In addition, palladium is a precious strategic resource with distinctive properties that is widely used in areas such as catalytic converters in vehicles and high-pressure turbine blades in jet engines [17,18]. Many scholars have investigated recovering valuable resources such as Pd in HLLW [19]. The long-lived radioisotope of Pd in HLLW is ^107^Pd, which has a low beta decay energy of 0.033 MeV and is essentially safe for most industrial applications [20]. Adsorbents such as hexacyanoferrate [14], nitrogen-donor-type adsorbents (R-Amine) [21], amino acid-functionalized cellulose microspheres [22], magnetic cross-linking chitosan nanoparticles [23], quaternary ammonium group ion exchange resin [24], bayberry tannin immobilized collagen fiber membranes [25], and ligand-immobilized meso-adsorbents [26] were studied to remove Pd. Among these adsorbents, hexacyanoferrate is expected because of its excellent adsorption capacity and easy accessibility [14,27]. Compared with expensive organic extractants, e.g., TODGA, costing CNY 150–500 (about USD 22–73) per gram, hexacyanoferrate is economical as a commercially available conventional reagent, costing CNY 0.1–0.3 (about USD 0.015–0.045) per gram.

Hexacyanoferrate powder is not suitable for the column, and a solid support is required for better surface area. SiO_2_ and PAN are commonly used as solid supports for these kinds of systems [28,29,30], as they have better mechanical and hydraulic properties, and a greater surface area. HLLW is a radioactive and heat-releasing material with high requirements for material irradiation resistance. Generally, inorganic adsorbents have better radiation resistance than that of equivalent organic solvent-extraction and ligand systems [31,32]. However, the effects of irradiation on hexacyanoferrate and palladium removal, despite being important for the industry, have rarely been discussed in existing research.

In this work, silica-based aluminum hexacyanoferrate was selected as the adsorbent. In addition, we study the γ-irradiation resistance and theoretical Pd(II) adsorption products of KAlFe(CN)_6_/SiO_2_. The KAlFe(CN)_6_/SiO_2_ adsorbent was prepared and irradiated with ^60^Co. The effects of different γ-irradiation doses on the crystal structure of adsorbent were analyzed. The adsorption performance of irradiated KAlFe(CN)_6_/SiO_2_ on Pd(II) at different HNO_3_ concentrations was investigated. Adsorption kinetics and adsorption isotherms before and after γ-irradiation were explored. Adsorption products with different adsorption rates and Pd(II) binding sites were calculated by using the DFT method, and their structural characteristics and free energy were compared.

## 2. Materials and Methods

### 2.1. Materials

Aluminum chloride hexahydrate (AlCl_3_∙6H_2_O) with 99% purity and potassium hexacyanoferrate trihydrate (K_4_Fe(CN)_6_∙3H_2_O) with 99.5% purity were purchased from Shanghai Macklin Inc, Shanghai, China. Palladium nitrate dihydrate (Pd(NO_3_)_2_·2H_2_O) with 99% purity was obtained from Beijing Innochem, Beijing, China. Nitric acid (HNO_3_) was obtained from Shanghai Sinopharm, Shanghai, China and was of analytical grade. Silica (SiO_2_) was purchased from Fuji Silysia Chemical Ltd., Kasugai, Japan. The conductivity of deionized water used in the experiments was less than 5 μS cm^−1^.

### 2.2. Preparation of KAlFe(CN)_6_/SiO_2_

The KAlFe(CN)_6_/SiO_2_ adsorbent was synthesized by using a two-step loading method. First, the silica was washed and dried at 90 °C for 1 day. The 1 M AlCl_3_ solution and 1 M K_4_Fe(CN)_6_ solution were successively sucked into the silica pores at a phase ratio of 10 cm^3^ g^−1^. Then, the filter flask was left at 25 °C for 1 day to fully react. When the solution had been layered and there was no newly generated precipitate, the product was removed, filtered, washed, and dried at 90 °C for 1 day. According to EDS mapping analysis, the KAlFe(CN)_6_ accounted for about 30% of the KAlFe(CN)_6_/SiO_2_ composite adsorbent in the mass.

### 2.3. Irradiation Treatment and Characterization

The KAlFe(CN)_6_/SiO_2_ adsorbent was γ-irradiated in an air atmosphere at 25 °C. The irradiation source was ^60^Co (10^5^ Ci), and the irradiation doses were 10, 50, 100, and 1000 kGy calibrated with a Frick dosing agent at a rate of 5 kGy/h. After irradiation, KAlFe(CN)_6_/SiO_2_ was characterized. Scanning electron microscopy (SEM) was performed with a Mira 3 (TESCAN ORSAY HOLDING a.s., Brno, Czech Republic). An X-ray diffractometer (XRD, D8 Advance, Brucker Corporation, Billerica, MA, USA) and a Fourier transform infrared spectrometer (FT–IR, Nicolet 6700, Thermo Fisher Scientific, Waltham, MA, USA) were used to analyze the crystal structure and chemical bonds of the adsorbents.

### 2.4. Batch Experiments

Batch experiments were conducted at 25 °C. In a clean vial, a Pd(II) solution (*V* = 5 mL) was mixed with KAlFe(CN)_6_/SiO_2_ (*m* = 0.05 g). The supernatant inside the vial was filtered and diluted. The concentration of Pd(II) in the filtrate was obtained by using an inductively coupled plasma (ICP) spectrometer (Shimadzu Corporation, Kyoto, Japan, ICP-7500).

The distribution coefficient of Pd(II) (*K*_d_) was calculated as follows:

(1) $$K_{d}=\left[\left(C_{0}-C_{e}\right)/C_{e}\right]\times \left(V/m\right),$$

where *C*_0_ is the concentration of Pd(II) in the preadsorption solution, *C*_e_ represents the equilibrium concentration, *V* is the volume of the Pd(II) solution, and *m* is the mass of the KAlFe(CN)_6_/SiO_2_ adsorbent.

The experimental adsorption capacity of Pd(II) (*Q*_e_) is defined as follows:

(2) $$Q_{e}=\left(C_{0}-C_{e}\right)\times \left(V/m\right).$$

### 2.5. DFT Calculations

In exploring the adsorption mechanism of Pd(II) by KAlFe(CN)_6_, the optimized structures and free energies of the adsorbent and adsorption products were calculated on the basis of DFT. Calculations were performed via VASP 6.2. The modeling and visualization of the hexacyanoferrates were conducted in VESTA [33]. Considering that the adsorption capacity may have been different, the Pd(II) products with adsorption rates of 100% (Pd[AlFe(CN)_6_]_2_) and 50% (Pd_0.5_K[AlFe(CN)_6_]_2_) were calculated. The crystal parameters and free-energy changes caused by the difference in Pd(II) adsorption sites in Pd_0.5_K[AlFe(CN)_6_]_2_ were compared. The generalized gradient approximation (GGA) method was selected as the wavefunction optimization potential. Calculations were performed in the inverted space by using the Perdew–Burke–Ernzerhof (PBE) functional [34].

## 3. Results and Discussion

### 3.1. XRD Analysis before and after Irradiation

**Figure 1:**
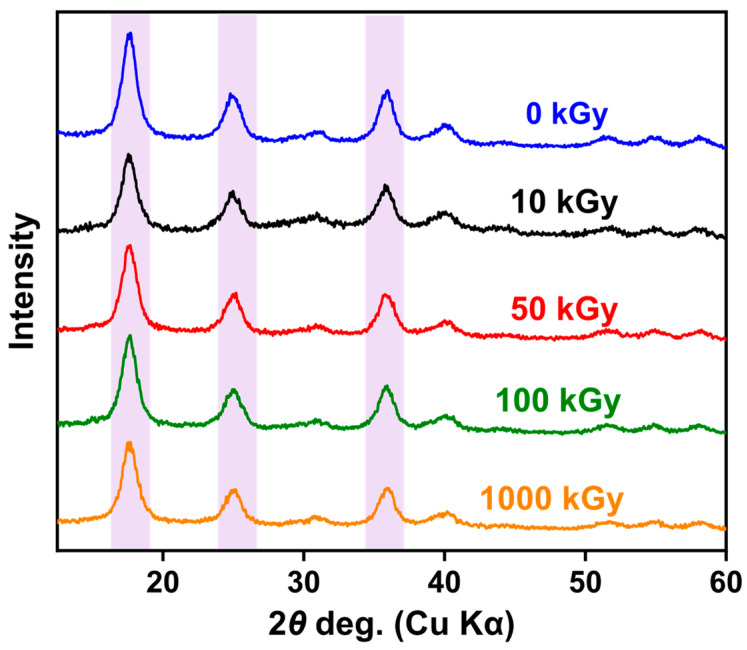
XRD patterns of KAlFe(CN)_6_ before and after irradiation.

In studying whether irradiation changes the crystal structure of KAlFe(CN)_6_, XRD characterization was performed on the KAlFe(CN)_6_ powder sample after irradiation (Figure 1). Table 1 shows the angle of incidence and lattice parameters of the KAlFe(CN)_6_ powder before and after irradiation.

As shown in Figure 1 and Table 1, the lattice of KAlFe(CN)_6_ expanded slightly after irradiation for 10, 50, 100, or 1000 kGy. The angles of the three main diffraction peaks slightly decreased with the increase in irradiation dose from 17.672°, 25.028°, and 36.051° to 17.482°, 24.840°, and 35.746°, respectively. Nevertheless, the strength and shape of the peaks in the spectrum remained similar throughout irradiation, indicating that irradiation did not cause a notably change in the substance, and the structure of the crystal remained stable. The results can be explained by the action mechanism of irradiation. The effect of γ rays is identical with that of β rays except for the thermal effect [35]. Under γ-ray irradiation, free electrons were formed in the KAlFe(CN)_6_ structure because of the photoelectric and Compton effects. The electrons interacted with the adsorbent and caused atomic displacements, forming defects such as dislocation loops [36,37].

Further analysis showed that, after irradiation at 1000 kGy, the lattice parameter of the KAlFe(CN)_6_ adsorbent increased slightly from 10.0155 to 10.0464 Å, and the unit cell remained face-centered cubic. Thus, the irradiation dose from 10 to 1000 kGy hardly affects the lattice structure and irregularity of KAlFe(CN)_6_, and the radiation resistance of the adsorbent was excellent.

### 3.2. SEM and FT–IR Analysis before and after Irradiation

**Figure 2:**
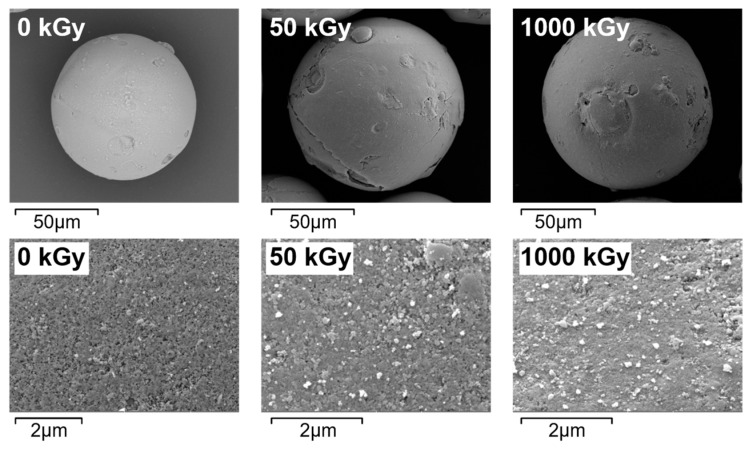
SEM images of KAlFe(CN)_6_/SiO_2_ before and after (left to right) 0, 50, and 1000 kGy irradiation.

The SEM images of KAlFe(CN)_6_/SiO_2_ before and after irradiation are shown in Figure 2. The adsorbent particle was spherical with a diameter of about 100 μm before irradiation. It maintained that shape and surface morphology with some minor cracks forming after 50 and 1000 kGy of γ irradiation. The adsorbents did not collapse or disintegrate after irradiation, indicating that KAlFe(CN)_6_/SiO_2_ had good radiation resistance.

**Figure 3:**
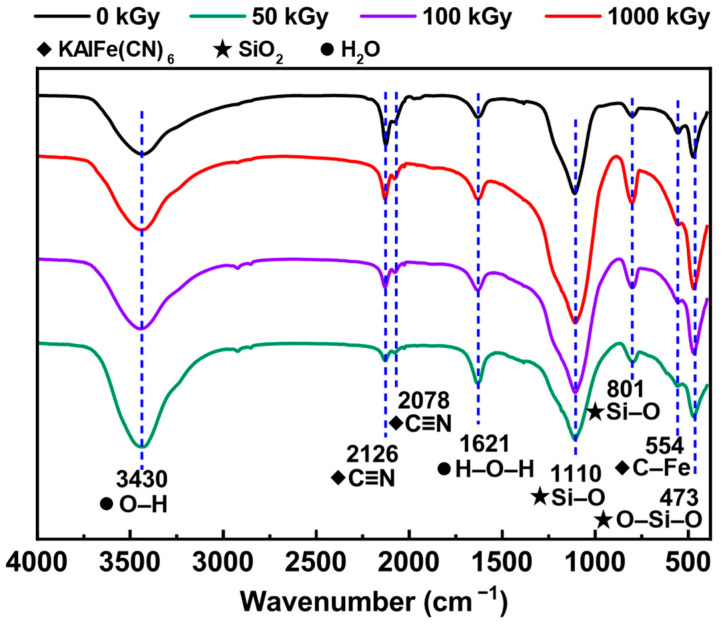
FT–IR patterns of KAlFe(CN)_6_/SiO_2_ before and after (top to bottom) 0, 50, 100, and 1000 kGy irradiation.

The FT–IR spectra of KAlFe(CN)_6_/SiO_2_ before and after irradiation are shown in Figure 3. The peak at 3430 cm^−1^ was attributed to the O–H stretching vibration peak, and 1621 cm^−1^ was the H–O–H bending vibration peak [38]; both came from crystal water. The peaks at 1110 and 801 cm^−1^ were assigned to the Si–O stretching vibration in SiO_2_, and 473 cm^−1^ corresponds to the inplane bending vibration of O–Si–O [38]. The peak at 554 cm^−1^ was assigned to the C–Fe stretching vibration [39]. The peaks at 2126 and 2078 cm^−1^ correspond to the C≡N stretching vibration [38,40], indicating the presence of hexacyanoferrate. The peak of CN^−^ weakened with the increase in irradiation dose, which indicates that the γ ray destroyed a minor portion of CN^−^ [41,42]. The above analysis shows that KAlFe(CN)_6_/SiO_2_ could preserve structural integrity under up to 1000 kGy of irradiation.

### 3.3. Adsorption Properties in Nitric Acid before and after Irradiation

**Figure 4:**
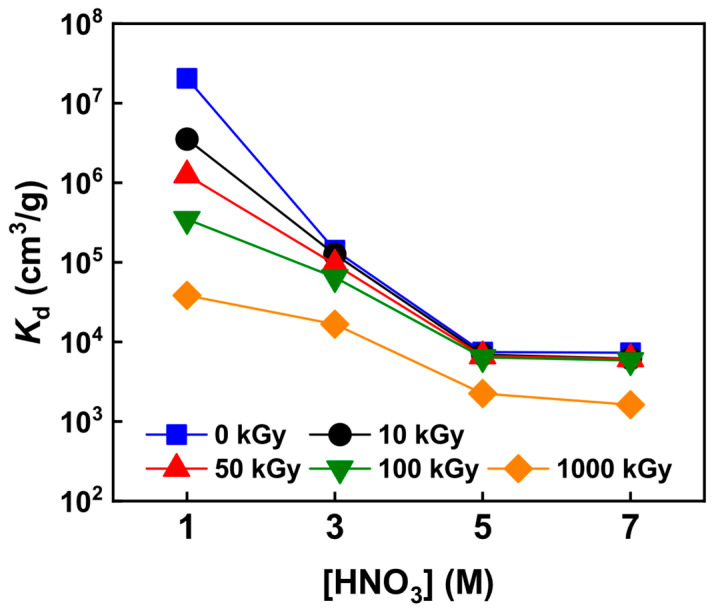
*K*_d_ of KAlFe(CN)_6_/SiO_2_ for Pd(II) before and after irradiation in 1–7 M HNO_3_; phase ratio = 100 cm^3^ g^−1^, [Pd] = 200 mg L^−1^, temperature = 25 °C, and contact time = 24 h.

The adsorption performance of the irradiated KAlFe(CN)_6_/SiO_2_ was investigated. Figure 4 shows the adsorption effects of different concentrations of HNO_3_ on Pd(II) after receiving 10 to 1000 kGy of irradiation. Distribution coefficient *K*_d_ was slightly reduced after irradiation, and the magnitude of the drop decreased with the increase in HNO_3_ concentration. In 5 and 7 M HNO_3_ concentration, *K*_d_ remained almost the same from 0 to 100 kGy. In 1 to 3 M HNO_3_ concentration, *K*_d_ was highly sensitive to the adsorption rate because the rate was high. In addition, *K*_d_ remained high after 1000 kGy of irradiation, ranging from 1625 to 38,445 cm^3^ g^−1^, indicating that the adsorption ability of the irradiated KAlFe(CN)_6_/SiO_2_ remained performant in 1 to 7 M HNO_3_.

**Figure 5:**
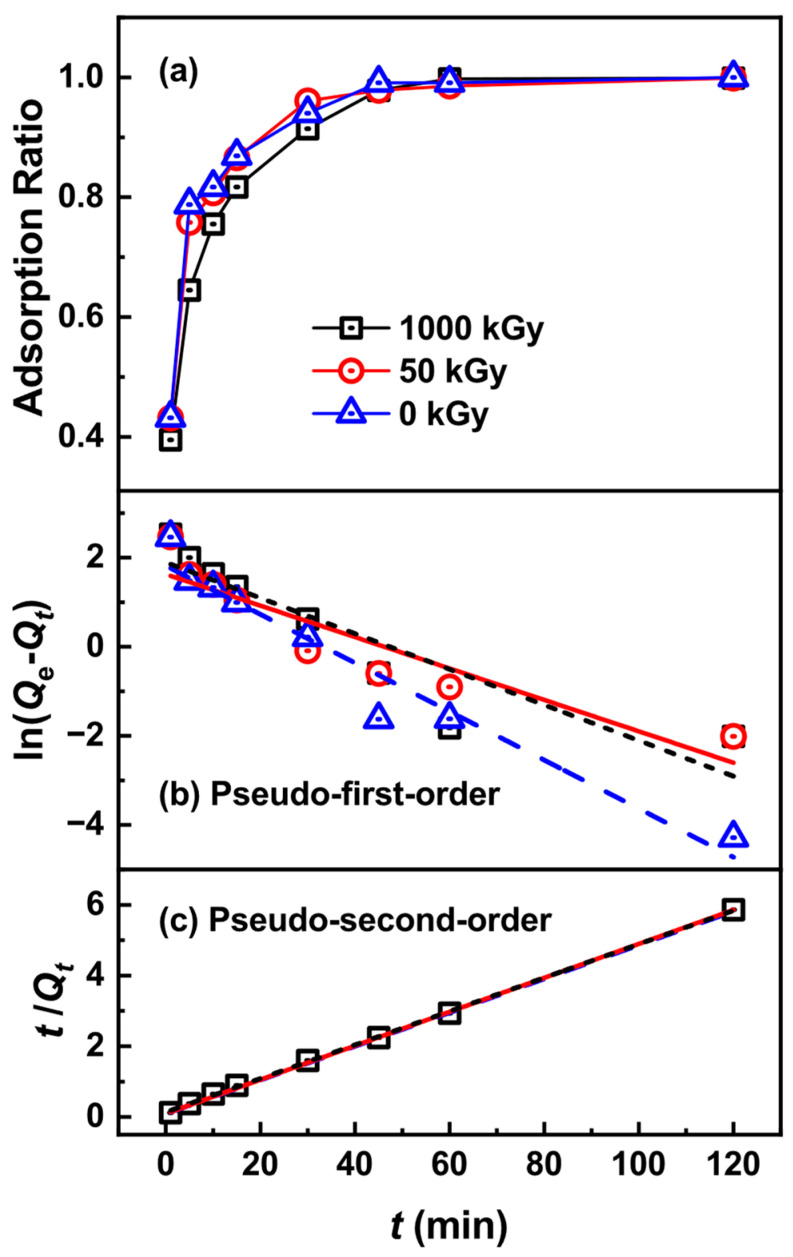
(**a**) Adsorption ratio of Pd(II) by KAlFe(CN)_6_/SiO_2_ versus adsorption time with a phase ratio = 100 cm^3^ g^−1^, [Pd] = 200 mg L^−1^, temperature = 25 °C, and contact time = 24 h; (**b**) pseudo-first-order adsorption kinetic model fitting; (**c**) pseudo-second-order adsorption kinetic model fitting.

The adsorption rate is an important factor in judging adsorption performance. Figure 5 shows the effects of contact time on Pd(II) adsorption. The adsorption ratios of the KAlFe(CN)_6_/SiO_2_ adsorbent for Pd(II) rapidly increased with time. When the contact time reached 15 min, the adsorption ratios of Pd(II) were over 87%, 86%, and 81% for 0, 50, and 1000 kGy irradiated adsorbents, respectively, indicating excellent adsorption kinetics. Subsequently, the adsorption rate of Pd(II) slowed down because the sites on KAlFe(CN)_6_/SiO_2_ tended to be saturated. After 45 min, the adsorption of Pd(II) by KAlFe(CN)_6_/SiO_2_ reached equilibrium, and the equilibrium adsorption rates were 99.9%, 99.8%, and 99.8%, respectively.

Further, the pseudo-first-order kinetic equation (Equation (3)) and pseudo-second-order kinetic equation (Equation (4)) were used for fitting to explore the adsorption rate control steps of KAlFe(CN)_6_/SiO_2_ on Pd(II) [43].

(3) $$ln\left(Q_{e}-Q_{t}\right)=ln\left(Q_{e}\right)-\left(k_{1}t\right),$$

(4) $$t/Q_{t}=1/\left(k_{2}Q_{e}^{2}\right)+t/Q_{e},$$

where *k*_1_ and *k*_2_ are fitted parameters related to the adsorption rate.

The pseudo-second-order equation fitted better than the pseudo-first-order equation (Figure 5). For the pseudo-first-order equation, the correlation coefficients were lower than 0.95; the values of the pseudo-second-order equation, on the other hand, were close to 1, indicating good linearity (Table 2). Moreover, the *Q*_e_ values of KAlFe(CN)_6_/SiO_2_ for Pd(II) obtained with the pseudo-second-order equation were 20.95, 20.82, and 20.61 mg g^−1^ for the irradiated adsorbents with 0, 50, and 1000 kGy, respectively, which values were close to the experimental data. *k*_2_ shows that the adsorption rate remained approximately equal before and after 50 kGy irradiation. These results indicate that the pseudo-second-order equation could describe the adsorption kinetics of KAlFe(CN)_6_/SiO_2_ well at different nitric acid concentrations, and the adsorption type of Pd(II) through the KAlFe(CN)_6_/SiO_2_ adsorbent was controlled via chemical adsorption.

### 3.4. Adsorption Isotherms before and after Irradiation

In investigating the capacity and mechanism of the adsorption of the KAlFe(CN)_6_/SiO_2_ adsorbent on Pd(II), adsorption isotherms were obtained from the experiments, and fitted using the Langmuir (Equation (5)) [44], Freundlich (Equation (6)) [45], and Redlich–Peterson (Equation (7)) [46] models.

(5) $$Q_{e}=\left(Q_{\max}K_{L}C_{e}\right)/\left(1+K_{L}C_{e}\right),$$

(6) $$Q_{e}=Q_{\max}C_{e}^{1/n},$$

(7) $$Q_{e}=\left(K_{R}C_{e}\right)/\left(1+K_{P}C_{e}^{g}\right),$$

where *Q*_max_ is the maximal adsorption capacity predicted by the model; *K*_L_, 1⁄*n*, *K*_R_, *K*_P_, and *g* are the fitting parameters.

**Figure 6:**
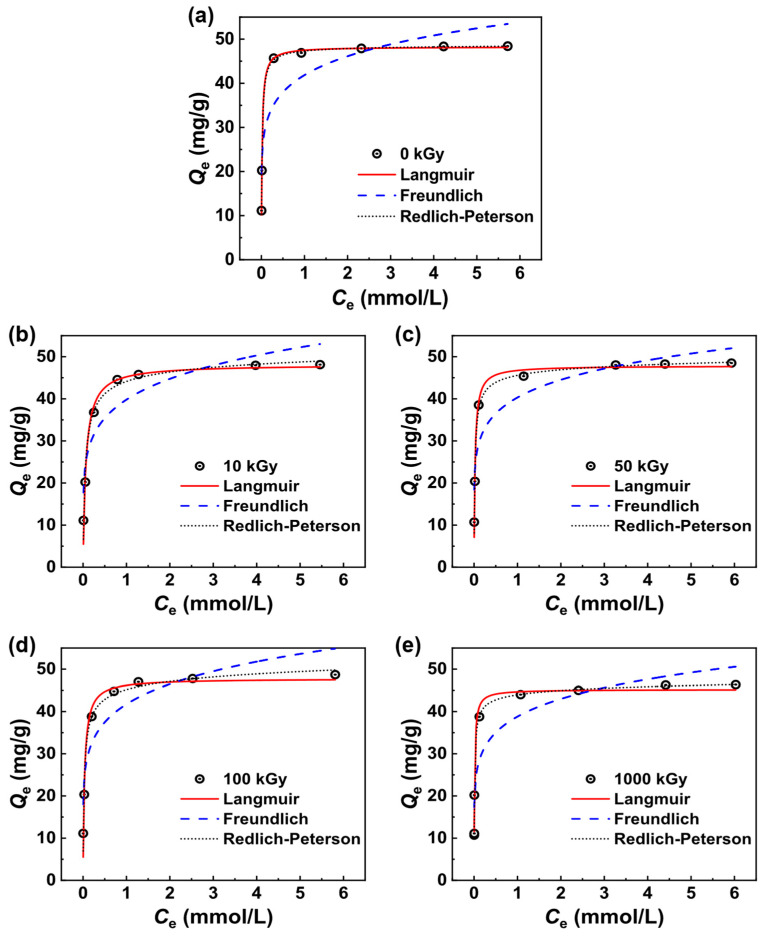
Adsorption isotherms and model fitting of KAlFe(CN)_6_/SiO_2_ for Pd(II) before and after irradiation in 3 M HNO_3_ with a phase ratio = 100 cm^3^ g^−1^, temperature = 25 °C, and contact time = 24 h. (**a**) before irradiation; after (**b**) 10 kGy, (**c**) 50 kGy, (**d**) 100 kGy, and (**e**) 1000 kGy irradiation.

As shown in Figure 6 and Table 3, the coefficients of determination *R*^2^ of the Langmuir and Redlich–Peterson models were higher than 0.96, and the fitted parameter *g* in the Redlich–Peterson model was between 0.96 and 0.995, indicating that the Redlich–Peterson model could be converted into the Langmuir model in these cases. With a smaller irradiation dose than 1000 kGy, the adsorption of KAlFe(CN)_6_/SiO_2_ on Pd(II) corresponded to the Langmuir model, suggesting that the adsorption sites on KAlFe(CN)_6_/SiO_2_ were on a single layer, and the adsorbed Pd(II) had no internal interaction [44].

The fitted results of Equation (5) show that, after irradiation with 10, 50, 100, and 1000 kGy, the maximal adsorption capacities *Q*_e_ of KAlFe(CN)_6_/SiO_2_ on Pd(II) were 48.1, 47.8, 47.7, and 45.1 mg g^−1^_,_ respectively. The relative decrease in the unirradiated sample was only 0.4%, 1.0%, 1.2%, and 6.2%, respectively. The results indicate that the adsorption capacity of KAlFe(CN)_6_/SiO_2_ was insignificantly influenced by the irradiation dose and remained virtually unchanged after no more than 100 kGy of irradiation. Thus, KAlFe(CN)_6_/SiO_2_ could be used for the separation and recovery of Pd(II) under radioactive conditions.

As shown in Table 4, compared with other adsorbents, the adsorption capacity of KAlFe(CN)_6_/SiO_2_ was higher with shorter equilibrium time.

### 3.5. DFT Calculations

**Figure 7:**
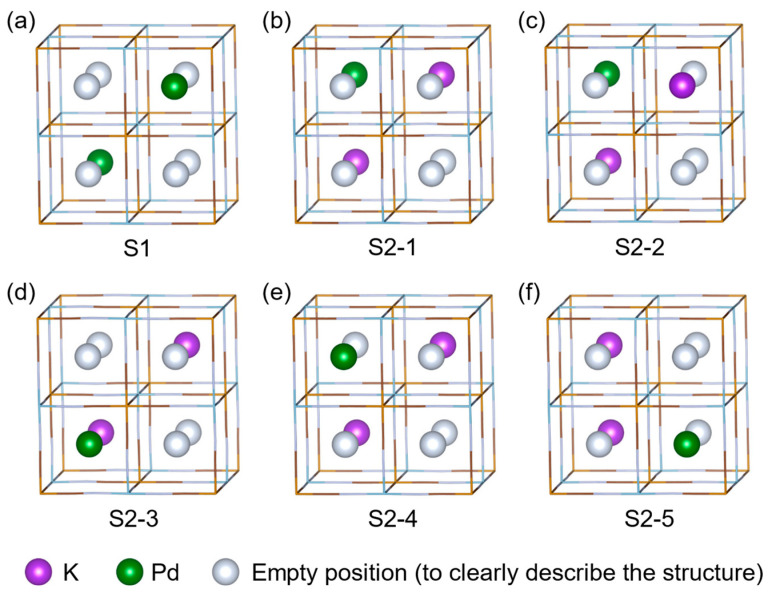
Optimized stable structures of (**a**) Pd[AlFe(CN)_6_]_2_ and (**b**–**f**) Pd_0.5_K[AlFe(CN)_6_]_2_.

The DFT method was applied to analyze the adsorption products of Pd(II) by KAlFe(CN)_6_. Stable structures with different adsorption rates and positions of adsorption sites were calculated. Figure 7a illustrates the optimized structure of the Pd[AlFe(CN)_6_]_2_ (S1) product of saturated adsorption. Figure 7b–f show the optimized structures of the Pd_0.5_K[AlFe(CN)_6_]_2_ product with an adsorption rate of 50%, namely, S2-1 to S2-5, for different adsorption sites. Table 5 lists the lattice parameters of these optimized structures, proving that all six types of products had FCC cells with similar volumes, where the cell of the saturated adsorption of product S1 was a cube, whereas unsaturated product S2 was a cuboid because of its asymmetry in the position of K and Pd atoms.

On the basis of the optimized stable structures before and after adsorption, the Helmholtz free energy (*F*) values were calculated. Thermodynamic calibration was performed via Phonopy [51] and VASPKIT [52], and the change in free energy (Δ*F*) was calculated as

(8) $$\Delta F=F_{{\mathrm{Pd}}_{m}K_{n}{\left[\mathrm{AlFe}{\left(\mathrm{CN}\right)}_{6}\right]}_{2}}+nF_{K^{+}}-2F_{\mathrm{KAlFe}{\left(\mathrm{CN}\right)}_{6}}-mF_{{\mathrm{Pd}}^{2+}},$$

where *F* represents the free energy, *m* is the number of Pd atoms, and *n* is the number of K atoms.

**Figure 8:**
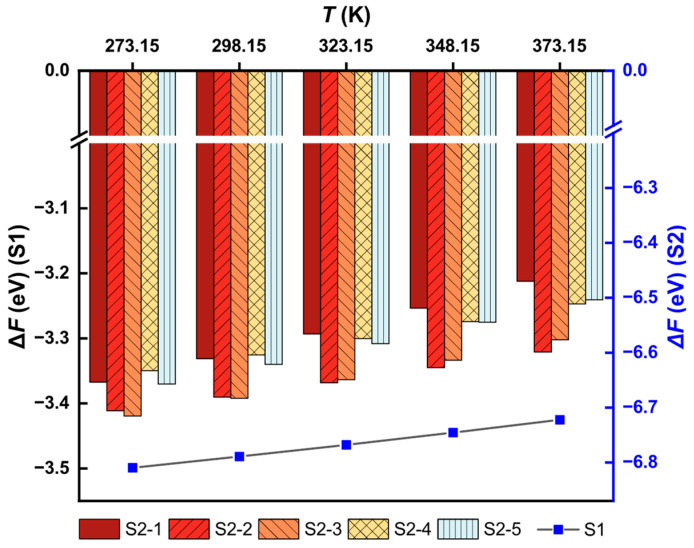
Calculated free-energy changes of different reactions.

The results are illustrated in Figure 8, where the free-energy changes of S1 are in the line chart and those of S2s are in the column chart. The Δ*F* of S1 was lower than that of S2, from 273.15 to 373.15 K, showing that S1 was spontaneously generated. The KAlFe(CN)_6_ adsorbent tended to completely absorb Pd(II) and become Pd[AlFe(CN)_6_]_2_.

S2 structures, on the other hand, were intermediate products of adsorption. The system free-energy changes indicate that S2-2 and S2-3 were likely generated more spontaneously than the others because of the low Δ*F*. In addition, the free-energy changes varied with temperature, that is, S2-3 was slightly superior, below 323.15 K, whereas S2-2 changed to the dominant structure of above 323.15 K.

## 4. Conclusions

The crystal structure and adsorption ability of gamma-irradiated KAlFe(CN)_6_/SiO_2_ on Pd(II) were investigated. SEM, XRD, and FT–IR characterizations showed that the peak positions and peak intensities of the irradiated adsorbent changed slightly, but its crystal structure was stable and not significantly damaged. After irradiation, the *K*_d_ of Pd(II) dropped slightly, and the magnitude of the drop decreased with the increase in HNO_3_ concentration. For the irradiation dose of 100 kGy, *K*_d_ on the adsorbent was higher than 5846 cm^3^ g^−1^, and *Q*_e_ was 48.1 mg g^−1^, which was only 1.2% lower than that of the unirradiated samples. The adsorption equilibrium was attained within 45 min, and the adsorption of Pd(II) followed the pseudo-second-order kinetic equation, which corresponds to chemical adsorption. The adsorption results indicated that KAlFe(CN)_6_/SiO_2_ achieved excellent adsorption performance on Pd(II) with 100 kGy irradiation. The DFT calculations showed that the stablest theoretical Pd(II) adsorption product of KAlFe(CN)_6_ was Pd[AlFe(CN)_6_]_2_, and this adsorption process was spontaneous. When the temperature was lower than 323.15 K, the most dominant intermediate product was Pd_0.5_K[AlFe(CN)_6_]_2_ with a cell volume of 1013.53 Å^3^, followed by Pd_0.5_K[AlFe(CN)_6_]_2_ with a cell volume of 1013.09 Å^3^. Therefore, KAlFe(CN)_6_/SiO_2_ could be used to separate and recover Pd(II) under radioactive conditions for use in other industries. Because of the low beta decay energy, the recovered Pd is essentially safe for industrial applications. Further study into recovering Pd from adsorbents is required, and there are several ongoing works on this topic.

## Figures and Tables

**Table 1:** Angles of incidence of main diffraction peaks and lattice parameters of KAlFe(CN)_6_ before and after irradiation.

Irradiation Dose (kGy)	Angle of Incidence 2*θ* (°)			Lattice Parameters *a* = *b* = *c* (Å)
	Peak 1	Peak 2	Peak 3	
0	17.672	25.028	36.051	10.0155
10	17.661	24.997	35.923	10.0246
50	17.642	24.958	35.884	10.0268
100	17.582	24.938	35.765	10.0323
1000	17.482	24.840	35.746	10.0464

**Table 2:** Adsorption kinetic parameters of Pd(II) by KAlFe(CN)_6_/SiO_2_ before and after irradiation.

Adsorption Kinetic Models	Parameters			
		0 kGy	50 kGy	1000 kGy
Pseudo-first-order kinetic model	*k*_1_ (10^−3^ mg g^−1^ min^−1^)	54.51 ± 5.06	35.26 ± 5.73	32.99 ± 7.34
	*Q*_e_ (mg g^−1^)	6.158 ± 1.29	5.015 ± 1.34	5.001 ± 1.46
	*R* ^2^	0.9427	0.8405	0.8038
Pseudo-second-order kinetic model	*k*_2_ (10^−3^ mg g^−1^ min^−1^)	25.36 ± 1.71	24.83 ± 1.83	17.48 ± 2.87
	*Q*_e_ (mg g^−1^)	20.95 ± 0.12	20.82 ± 0.10	20.61 ± 0.19
	*R* ^2^	0.9998	0.9999	0.9994
Experimental data	*Q* (mg/g)	20.60 ± 0.8	20.48 ± 0.8	20.45 ± 0.8

**Table 3:** Fitted parameters of adsorption isotherms before and after irradiation.

Isotherm Models	Parameters	Irradiation Dose (kGy)				
		0	10	50	100	1000
Langmuir model	*Q*_max_ (mg g^−1^)	48.3 ± 0.3	48.1 ± 1.5	47.8 ± 0.9	47.7 ± 1.4	45.1 ± 1.2
	*K*_L_ (L mg^−1^)	60.8 ± 2.5	15.5 ± 3.2	41.9 ± 5.9	28.9 ± 6.5	82.2 ± 13.6
	*R* ^2^	0.9989	0.9724	0.9685	0.9707	0.9769
Freundlich model	*Q*_max_ (mg g^−1^)	41.8 ± 2.8	39.8 ± 2.3	40.3 ± 2.4	41.7 ± 2.4	38.8 ± 2.5
	1/*n*	0.14 ± 0.04	0.17 ± 0.04	0.14 ± 0.03	0.16 ± 0.03	0.15 ± 0.03
	*R* ^2^	0.8305	0.8683	0.8698	0.8686	0.8627
Redlich–Peterson model	*K*_R_ (L mg^−1^)	3001.8 ± 14.2	976.8 ± 30.8	2474.7 ± 48.0	1922.4 ± 57.8	4119.1 ± 81.1
	*K*_P_ (L mg^−1^)	62.43 ± 3.18	21.1 ± 7.4	53.3 ± 11.3	41.6 ± 13.3	92.7 ± 19.8
	g	0.995 ± 0.006	0.96 ± 0.04	0.972 ± 0.018	0.96 ± 0.03	0.98 ± 0.02
	*R* ^2^	0.9991	0.9775	0.9909	0.9801	0.9814
Experimental data	*Q*_e_ (mg g^−1^)	48.4 ± 0.8	48.2 ± 0.8	48.1 ± 0.8	47.8 ± 0.8	45.5 ± 0.8

**Table 4:** Comparison of adsorption capacity and equilibrium time on Pd(II) with different adsorbents.

Adsorbent	Solution	*Q*_e_ (mg g^−1^)	*T*_e_ * (min)	Reference
Thiourea–formaldehyde resin	pH = 4	31.9	–	[47]
Bayberry tannin immobilized collagen fiber	pH = 4	33.4	240	[25]
PMA–SNP	pH = 3	53.6	480	[48]
Commercial activated carbon pellets	2 M HCl	27.2	–	[49]
CA–BOPhen@SiO_2_–P	3 M HNO_3_	35.0	10	[50]
K_2_NiFe(CN)_6_/SiO_2_	3 M HNO_3_	41.0	60	[14]
KAlFe(CN)_6_/SiO_2_	3 M HNO_3_	48.3	45	This work

* Equilibrium time.

**Table 5:** Structural parameters of Pd[AlFe(CN)_6_]_2_ and Pd_0.5_K[AlFe(CN)_6_]_2_ with different adsorption positions.

Structure	*a* (Å)	*b* (Å)	*c* (Å)	*α* = *β* = *γ* (°)	*V* (Å^3^)
S1	10.0347	10.0347	10.0347	90	1010.44
S2-1	10.0375	10.0375	10.0421	90	1011.75
S2-2	10.0415	10.0466	10.0466	90	1013.53
S2-3	10.0440	10.0432	10.0432	90	1013.09
S2-4	10.0297	10.0327	10.0327	90	1009.54
S2-5	10.0617	10.0617	10.0531	90	1017.76

## Data Availability

Not applicable.
